# Foreseen Effects of Climate-Impacted Scenarios on the Photochemical Fate of Selected Cyanotoxins in Surface Freshwaters

**DOI:** 10.1021/acs.est.1c03440

**Published:** 2021-08-03

**Authors:** Davide Vione, Fernando L. Rosario-Ortiz

**Affiliations:** † Dipartimento di Chimica, Università degli Studi di Torino, Via Pietro Giuria 5, 10125 Torino, Italy; ‡ Department of Civil, Environmental, and Architectural Engineering, University of Colorado Boulder, 1111 Engineering Drive, 428 UCB, Boulder, Colorado 80309, United States; § Environmental Engineering Program, University of Colorado Boulder, Boulder, Colorado 80309, United States

**Keywords:** Microcystin-LR, Cylindrospermopsin, Sensitized phototransformation, Summer stratification, Water browning, Evaporative concentration, Extended drought periods

## Abstract

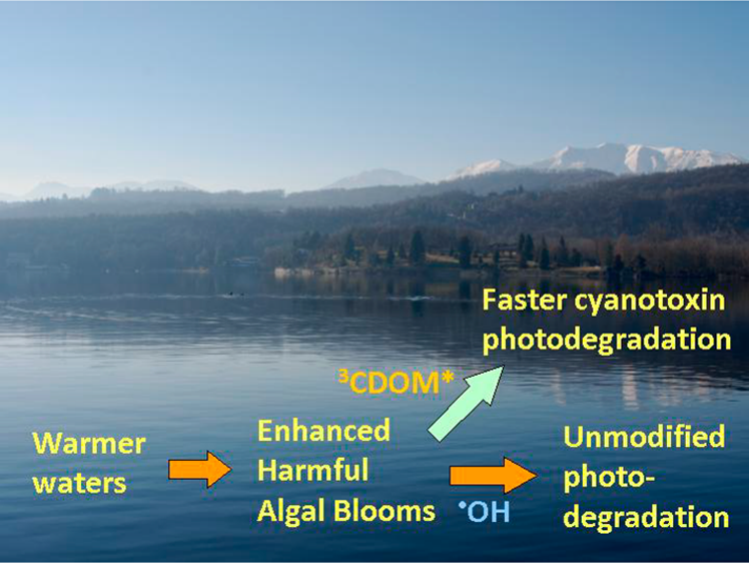

Cyanobacteria populate most water environments, and their ability to effectively exploit light and nutrients provide them with a competitive advantage over other life forms. In particular conditions, cyanobacteria may experience considerable growth and give rise to the so-called harmful algal blooms (HABs). HABs are often characterized by the production of cyanotoxins, which cause adverse effects to both aquatic organisms and humans and even threaten drinking water supplies. The concentration of cyanotoxins in surface waters results from the budget between production by cyanobacteria and transformation, including photodegradation under sunlight exposure. Climate change will likely provide favorable conditions for HABs, which are expected to increase in frequency over both space and time. Moreover, climate change could modify the ability of some surface waters to induce phototransformation reactions. Photochemical modeling is here carried out for two cyanotoxins of known photoreaction kinetics (microcystin-LR and cylindrospermopsin), which follow different phototransformation pathways and for particular freshwater scenarios (summertime stratification in lakes, water browning, and evaporative water concentration). On this basis, it is possible to quantitatively predict that the expected changes in water-column conditions under a changing climate would enhance photodegradation of those cyanotoxins that are significantly transformed by reaction with the triplet states of chromophoric dissolved organic matter (^3^CDOM*). This is known to be the case for microcystin-LR, for which faster photodegradation in some environments would at least partially offset enhanced occurrence. Unfortunately, very few data are currently available for the role of ^3^CDOM* in the degradation of other cyanotoxins, which is a major knowledge gap in understanding the link between cyanotoxin photodegradation and changing climate.

## Abstract 2

Climate change is expected to enhance both the production of cyanotoxins in surface waters and, at least in some cases, their photodegradation.

## 1 Introduction

Cyanobacteria are organisms that trace their origins to billions of years into the past, and they are ubiquitous in all aquatic environments. Cyanobacteria can undergo rapid growth under favorable conditions, which may result in what is called harmful algal blooms (HABs) events. These events, with frequencies that are impacted by both anthropogenic activities (e.g., enhanced nutrient inputs into reservoirs) and climatic factors,^1^ result in numerous concerns, from damage to aquatic organisms to potential impacts to human health and animals, via exposure through recreation or potentially through potable water.^2^

The growth of cyanobacteria in water bodies is affected by water temperature, light availability, mixing vs stratification conditions, and the presence of nutrients.^3^ Different species of cyanobacteria are able to exploit to their advantages to the variable conditions that can be found in surface waters. In fact, some cyanobacteria grow at the water surface where radiation is particularly intense, and they tend to bloom during summer stratification of lake water,^4−6^ while others prefer conditions of low radiation intensity that can be found in turbid waters, for example, during lake overturn.^7^ Cyanobacteria of different species also take advantage of variable levels of nutrients. Elevated values of phosphorus are usually favorable to cyanobacteria blooms; some species can also fix atmospheric nitrogen (e.g., *Anabaena flos-aquae*)^8^ and grow well in hypertrophic water bodies that are rich in phosphorus but where dissolved nitrogen is usually the limiting element for most living organisms.^9^ At the same time, the ability of cyanobacteria to quickly assimilate phosphorus allows them to grow even in oligotrophic and mesotrophic environments, where the supply of this element is low and often irregular in time.^10,11^

Climate change is causing several modifications to environmental conditions, many of which are or can become favorable to the growth of cyanobacteria. Warmer water during summer and a longer period of thermal stratification in lakes can favor the blooms of some organisms (e.g., *Microcystis*), as well as their likelihood to induce HABs.^4,5,12,13^ Climate change is also increasingly characterized by alternations of drought periods and floods that deeply alter the hydrology of water basins and enhance the mobilization of nutrients.^14−17^ As mentioned above, the availabilities of nitrogen and phosphorus, and an intermittent supply for the latter, are conditions that either prove favorable to the growth of cyanobacteria or provide these species with a competitive advantage over other living organisms.

For the above reasons, surface freshwaters in the future might experience an increase in both the overall cyanobacteria blooms and the toxin-producing blooms, with a predicted higher occurrence of cyanotoxins in water environments.^18^ Both the environmental and human health impacts of cyanotoxins will have to be considered,^19^ as well as their transformation and fate in surface waters.^20,21^ A summary of the main cyanotoxins and the genera producing them is provided in Table 1.^22,23^ The fate of cyanotoxins in surface waters depends on both biotic and abiotic processes, with half-lives in the order of hours to weeks,^20,24^ with photochemical processes potentially having faster kinetics compared to what would be observed for a biological process.

**Table 1:**
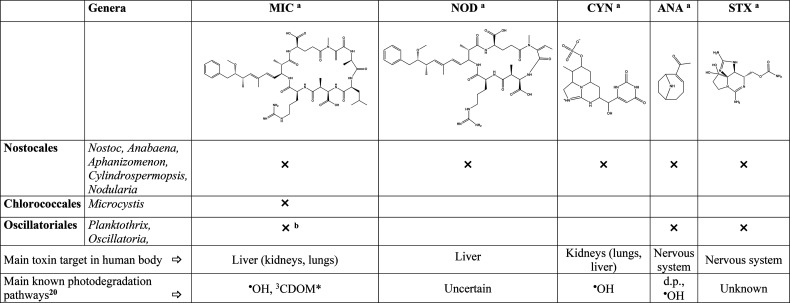
Summary of Main Families of Cyanobateria and of the Most Relevant Cyanotoxins That Can Be Produced in Surface Waters during HABs*

* The symbol “×” means that the given toxin is produced by the cyanobacteria under consideration. The main target organs in the human body and the main photodegradation pathways (when known) of each family of toxins are also provided.^20,22,23^^•^OH, hydroxyl radical; ^3^CDOM*, excited triplet states of chromophoric dissolved organic matter; d.p., direct photolysis.

a MIC, microcystins; NOD, modularins; CYN, cylindrospermopsin; ANA, anatoxins; STX, saxitoxins. The reported structures refer to microcystin-LR, nodularin, cylindropermopsin, anatoxin-a, and saxitoxin, respectively.

b MIC are produced by Oscillatoriales in small amounts compared to ANA and STX.

The possible impact of climate change on photochemical processes might thus play an important role in the future evolution of cyanotoxin concentration in surface waters. Some environments are expected not to undergo important modifications in photochemistry,^25^ which could thus not offset higher HAB occurrence. In other cases, the impacts of climate change on the chemistry and hydrology of some surface freshwaters are expected to significantly affect photochemical transformations.^26^ Here, we consider some scenarios where the photochemical effects of climate change can be quantitatively predicted, at least as a first approximation, as well as two cyanotoxins (microcystin LR, cylindrospermopsin) for which photoreaction parameters are known well enough to allow for quantitative assessments.^20^ Model predictions enable the identification of possible future trends in photodegradation kinetics and of key knowledge gaps, which prevent a clear understanding of how the fate of other cyanotoxins may be impacted by a warming future.

## 2 Summertime Thermal Stratification in Lake Water

Summers that become warmer because of climate change will favor thermal stratification in lakes.^27^ During stratification, surface and warm waters remain cut off from circulating and mixing with bottom waters, allowing for changes in chemical and biological processes. Longer stratification of lake water means, on the one hand, that more time is available for several species of cyanobacteria to grow and eventually produce toxins.^12,13^ On the other hand, it also provides more time for sunlight to induce toxin photodegradation^20,21^ by both direct and sensitized processes,^26,28^ the latter including the formation of different reactive intermediates from optically active species (most importantly, dissolved organic matter and inorganic nitrogen). The photochemical degradation processes usually follow pseudo-first-order kinetics, with a degradation rate proportional to the concentration of the compound being degraded and with a fixed half-life time, which is the time needed to halve the compound’s concentration.^29,30^

**Figure 1:**
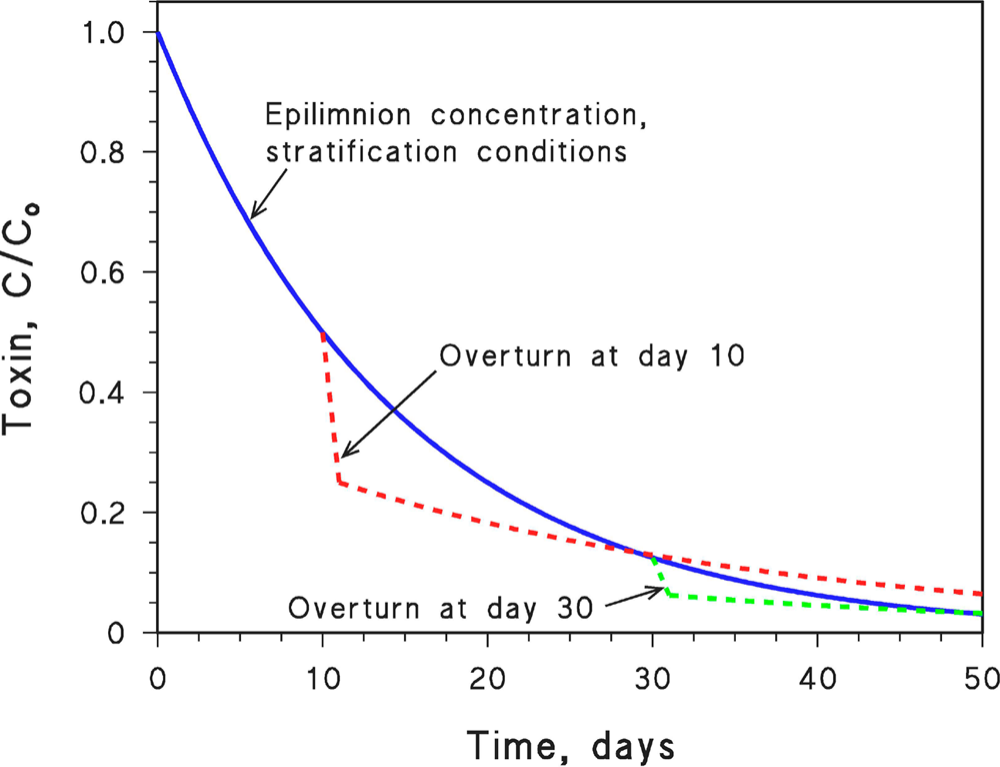
Simplified time trends of the concentration of a cyanobacterial toxin in the epilimnion of a stratified lake, due to photodegradation with a half-life time (*t*_1/2_) of 10 days (first-order degradation rate constant *k*_d_ = 0.069 day^–1^), as per microcystin-LR under reasonably favorable conditions (midlatitude summertime, taking the day–night cycle into account, *vide infra*). The dashed curves were obtained under the assumption that the lake underwent overturn at day 10 or at day 30. As a consequence of overturn, it was further assumed that the epilimnion was diluted with an equal volume of toxin-free water and that the subsequent photodegradation kinetics in the whole lake volume was halved (*k*_d_ = 0.035 day^−1^, *t*_1/2_ = 20 days) compared to that observed in the epilimnion before overturn.

It is hypothesized here that a HAB develops in the surface water layer (epilimnion) of a stratified lake; then, most cells die and release the toxin into the lake water. We assume that one has an initial concentration of toxin in the epilimnion, while the hypolimnion (the deep layer of the stratified lake) is toxin free. Compared to a mixing lake, a stratified lake experiences enhanced photodegradation in the epilimnion which is better illuminated by sunlight than the whole water column.^31^ Therefore, the longer the stratification is, the more efficient is cyanotoxin photodegradation in the epilimnion. Eventual lake overturn distributes the cyanotoxin in the whole lake volume, quickly decreasing the concentration in the epilimnion, increasing the concentration in the hypolimnion, and slowing cyanotoxin photodegradation (Figure 1). Additional assumptions were here made to simplify calculations: (i) The lake is a stationary system where water residence time is much longer than the time scale of photochemical reactions, which well applies to large lakes with limited water inflow or outflow. (ii) Virtually all of the incident radiation is absorbed in the epilimnion, so that the hypolimnion is in the dark. This is reasonable in many systems, considering that photochemically active radiation (300–500 nm) has a shorter wavelength and thus penetrates even less than photosynthetically active radiation in the water column. Furthermore, the presence of abundant solid material in suspension (i.e., dead cells) causes scattering phenomena that increase the optical path length of radiation in water,^32^ thereby enhancing absorption in an even shallower surface layer. (iii) At overturn, the epilimnion is diluted with an equal volume of toxin-free hypolimnion water. The combination of assumptions (ii, iii) ensures that the kinetics of toxin photodegradation is halved in the mixing lake compared to the epilimnion during stratification (at overturn, the same radiation is absorbed but the volume in which photoreactions take place is doubled).

It is shown in Figure 1 that early overturn (dashed curves) would slow cyanotoxin photodegradation but also cause a quick decrease of the concentration of cyanotoxin at the water surface, due to dilution by toxin-free deep water. Of course, there is an equally fast increase of toxin concentration in deep water. In contrast, longer stratification as could be caused by climate change (solid curve) would keep the toxin in the epilimnion, where it undergoes effective photodegradation and maintains the hypolimnion toxin free until water overturn. Figure 1 shows that it would take 20 days (i.e., *t* = 2 *t*_1/2_, where *t*_1/2_ = 10 days is the lifetime in the epilimnion) for faster photodegradation to compensate for concentration decrease (plus slower photodegradation) at overturn.

Longer stratification/late overturn clearly modify the overall lake conditions, with important effects on toxin concentration and photodegradation kinetics. Actual ecological consequences may be variable depending on the impact of different toxin concentrations in different environments (epilimnion vs hypolimnion), the use of lake water by human activities (e.g., recreation or drinking water), the depth at which water is taken up from the lake, if applicable, and whether or not the hypolimnion becomes anoxic during water stratification. If the hypolimnion maintains sufficient oxygen to host fish and other aerobic life forms, these could escape from toxic water at the surface in the stratification scenario.

## 3 Summer Stratification and Water Browning

In some environments, climate change might affect summer stratification in an additional way. Increased precipitation, or an increased frequency of extreme rain events, can enhance the export of organic matter from soil to surface waters, thereby increasing both the content of dissolved organic carbon (DOC) and that of the chromophoric dissolved organic matter (CDOM). Lake water will thus become darker and more carbon rich, the first effect being more immediately evident and lending its name to the phenomenon (water *browning* or *brownification*).^33,34^ Strong precipitation events also enhance export of nutrients from the basin to the water bodies,^16,17^ which can favor algal growth.

Compared to the original lake water, brownified water is darker and less conducive to the penetration of sunlight, which is thus able to heat up only a smaller fraction of the lake volume. This phenomenon affects thermal stratification because water browning causes the epilimnion to become shallower.^35^ Browning is expected to mostly affect relatively large lakes with long water residence times, where the DOC concentration is currently lower (on average) compared to smaller lakes.^34^

In a Lambert–Beer approximation, the spectral photon flux density of sunlight at the wavelength λ and depth *d* (*p*(λ,*d*)) can be expressed as follows:^36^
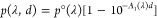
1where *p*°(λ) is the spectral photon flux density at the water surface, and *A*_1_(λ) is water absorbance at unit depth. As a first approximation, one has *A*_1_(λ) = A_o_ DOC e^–*S* λ^,^36^ where A_o_ is a proportionality factor, and *S* is the spectral slope. By considering this in eq 1, one has that light penetration in water depends on the product DOC × *d*. Water browning increases the DOC value (as well as the CDOM content), thereby decreasing at the same time the depth of the eplilimnion, *d*_epi_.^35,37^ Again as a first approximation, one might assume that the gradual increase (year after year) of the DOC value would affect *d*_epi_, so that the product DOC × *d*_epi_ remains constant.

**Figure 2:**
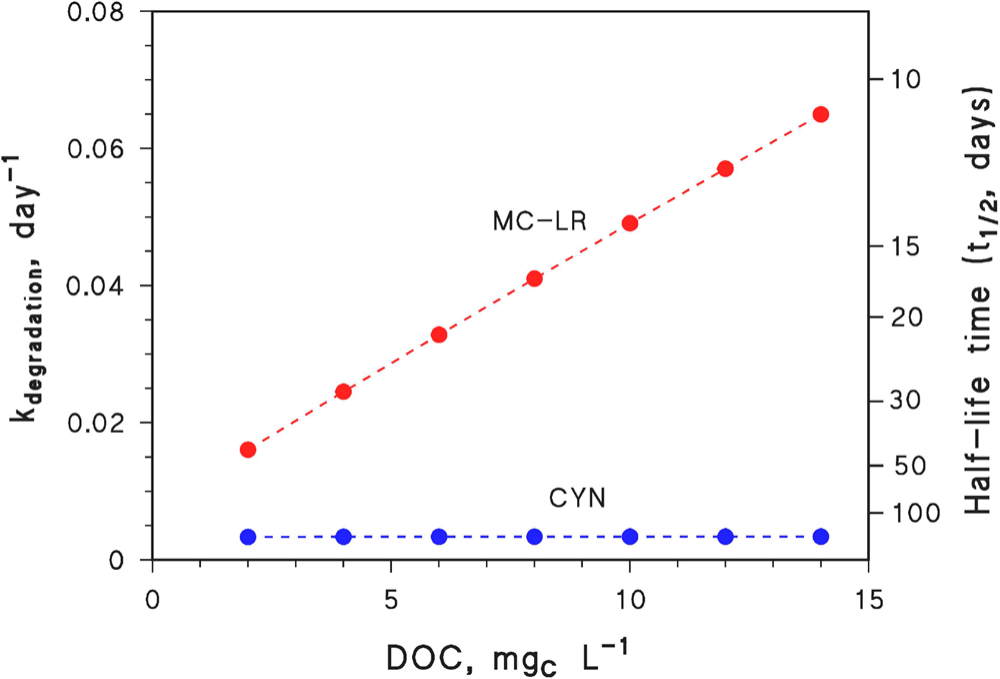
Modeled photodegradation kinetics (left *Y*-axis, first-order rate constants; right *Y*-axis, half-life times) of MC-LR and CYN, as a function of the DOC value of water, assuming constant DOC × *d*_epi_ = 30 m mg_C_ L^–1^. Other water conditions: 10^–4^ mol L^–1^ NO_3_^–^, 10^–6^ mol L^–1^ NO_2_^–^, 10^–3^ mol L^–1^ HCO_3_^–^, and 10^–5^ mol L^–1^ CO_3_^2–^. Photochemical modeling was carried out with the APEX software (Aqueous Photochemistry of Environmentally occurring Xenobiotics).^31^ Note that *t*_1/2_ = 0.693 *k*^–1^.

To see how this phenomenon might affect cyanotoxin photodegradation, we consider two different compounds with rather well-known degradation kinetics and pathways. Also note that we are only considering CDOM, nitrate, and nitrite as sensitizers, given the limited work on the potential for other algae components to sensitize photochemical degradation. Microcystin-LR (MC-LR) has a known second-order reaction rate constant with ^•^OH, *k*_MC-LR+^•^OH_ = 1.13 × 10^10^ L mol^–1^ s^–1^.^38^ However, it is known from laboratory irradiation experiments (cm-range water depth) that reaction with ^•^OH accounts for only ∼15% of the overall photodegradation of MC-LR, while the rest is accounted for by reactions with triplet state CDOM (^3^CDOM*).^20,39^ Given the experimental conditions,^39^ this datum is consistent with a second-order reaction rate constant with ^3^CDOM* around *k*_MC-LR+^3^CDOM*_ = 1.5 × 10^9^ L mol^–1^ s^–1^. Note that this rate constant is expressed in relationship to the reaction rate constant between ^3^CDOM* and 2,4,6-trimethylphenol.^40,41^ In contrast, cylindrospermopsin (CYN) undergoes degradation mainly by reaction with ^•^OH, with a second-order reaction rate constant *k*_CYN+^•^OH_ = 5 × 10^9^ L mol^–1^ s^–1^.^42,43^ There is evidence that the reaction of CYN with ^3^CDOM* is not important^21^ (for most other cyanotoxins the importance of this reaction is simply not known), while neither CYN nor MC-LR undergo significant direct photolysis.^20^ The photodegradation kinetics of the two compounds in an epilimnion with increasing DOC and decreasing depth, as expected in the case of browning waters (assuming constant DOC × *d*_epi_), is reported in Figure 2. It should be remarked that CYN is also known to react significantly with CO_3_^•–^,^44^ which is mainly formed upon oxidation of HCO_3_^–^ and CO_3_^2–^ by ^•^OH.^45^ The degradation of CYN by CO_3_^•–^ is expected to slow considerably with increasing DOC in the conditions of Figure 2 because the process is very efficiently inhibited by dissolved organic matter.^37,44^

The increasing photodegradation kinetics of MC-LR in the epilimnion with increasing DOC and decreasing *d*_epi_, as shown in Figure 2, would be accounted for by enhanced reaction with ^3^CDOM* because the steady-state [^3^CDOM*] in such conditions increases with increasing DOC.^37^ In contrast, [^•^OH] would remain almost constant in the epilimnion because enhanced ^•^OH scavenging by increasing DOC would be offset by decreasing *d*_epi_ (that is, ^•^OH concentrations in the epilimnion in this case are practically independent of the DOC).^37^ This consideration accounts for the absence of important modifications in the predicted photodegradation kinetics of CYN. The latter is also quite slow, coherently with model predictions about the behavior of compounds that mostly react with ^•^OH in these environments.^37^ In contrast, browning could lead to an important enhancement of phototransformation, in the epilimnion, of compounds that like MC-LR react with ^3^CDOM* to a significant extent.

## 4 Evaporative Water Concentration

**Figure 3:**
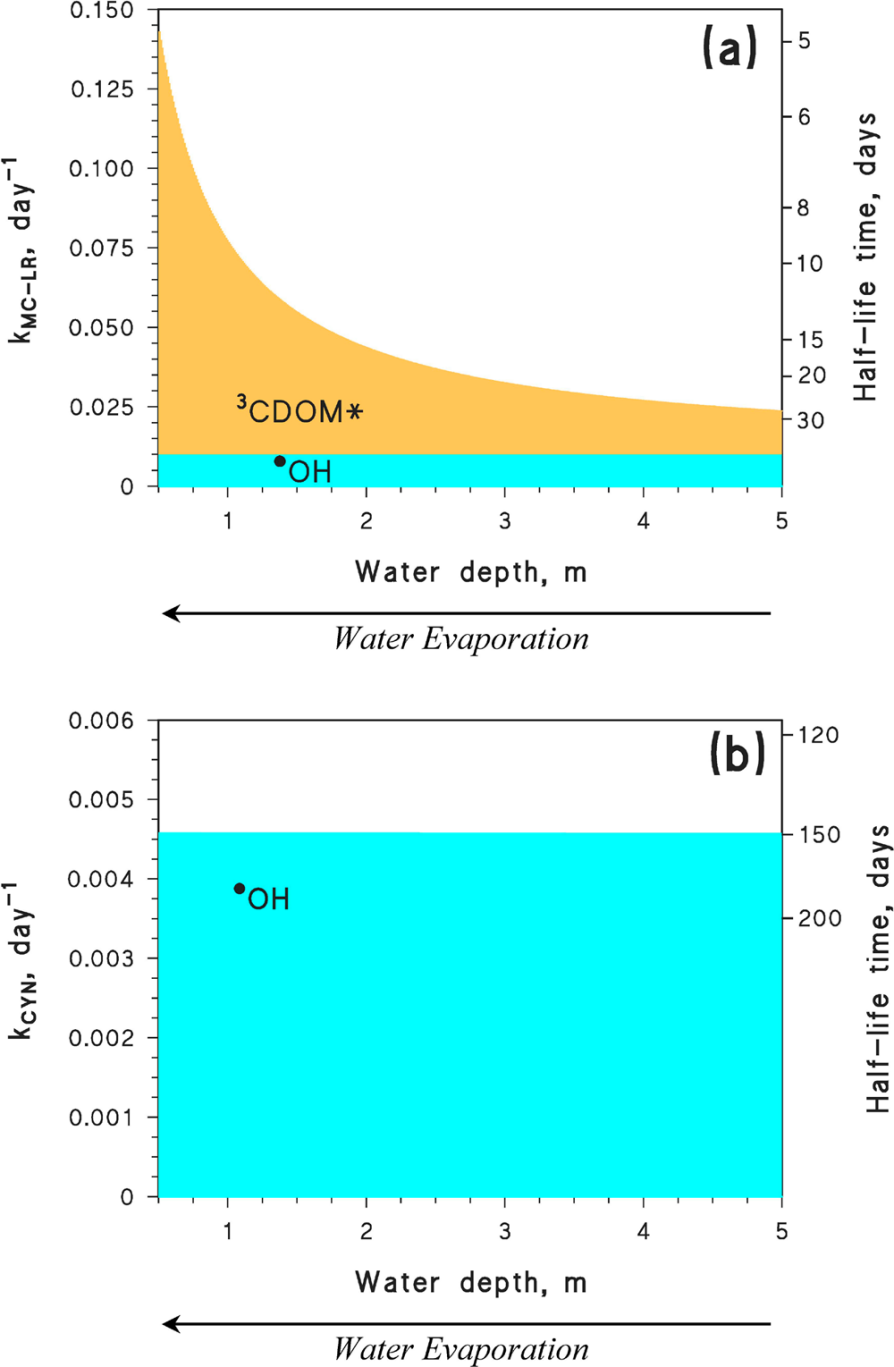
Modeled photodegradation kinetics (rate constants and half-life times, with *t*_1/2_ = 0.693 *k*^–1^) of MC-LR (a) and CYN (b) in lake water undergoing the phenomenon of evaporative concentration. The color code highlights the different photochemical reaction pathways. Initial water conditions (*d* = 5 m): 4 mg_C_ L^–1^ DOC, 2 × 10^–4^ mol L^–1^ NO_3_^–^, 2 × 10^–6^ mol L^–1^ NO_2_^–^, 2 × 10^–3^ mol L^–1^ HCO_3_^–^, and 2 × 10^–5^ mol L^–1^ CO_3_^2–^. Simulations were carried out with the APEX software.^31^ By comparison, note that during the *Millennium drought* the average water depth in the Australian Lower Lakes decreased from 2.4 to 1.2 m (Lake Alexandrina) and from 1.5 to 0.5 m (Lake Albert).^48^

The irregular precipitation regime that might be experienced in many regions of the world, as a consequence of climate change, might easily produce conditions where extended periods of drought are abruptly ended by heavy rainfall.^14,15^ During drought periods, water scarcity combined with intense heat might produce the phenomenon of evaporative water concentration. According to this phenomenon, which has for instance been observed in the Australian Lower Lakes during the so-called *Millennium drought*,^46,47^ water is lost, but nonvolatile solutes are not, which causes an increase in both salinity and the concentration values of most solutes. These changes in water chemistry and depth have interesting implications for photochemical transformation processes,^26,48^ which are amenable to photochemical modeling. By taking again the behavior of MC-LR and CYN into account, the implications of evaporative water concentration for cyanotoxin photodegradation are reported in Figure 3. The photodegradation kinetics of MC-LR is shown to be considerably enhanced by water evaporation because of the acceleration of degradation by ^3^CDOM*. In contrast, the reaction kinetics with ^•^OH is not modified significantly by evaporative concentration.

The reason is that, in the case of ^•^OH, water concentration enhances both the ^•^OH sources (NO_3_^–^, NO_2_^–^, CDOM) and the ^•^OH sinks (mostly dissolved organic matter, DOM). By proportionally increasing the rates of both ^•^OH formation and scavenging, the steady-state [^•^OH] remains unaltered.^48^ In contrast, while CDOM is the ^3^CDOM* source, the only important ^3^CDOM* sink is represented by dissolved O_2_. Because CDOM undergoes evaporative concentration but volatile O_2_ does not, water evaporation increases the concentration of ^3^CDOM* sources but not that of the scavengers. Therefore, the overall result is an enhancement of [^3^CDOM*] and of the related processes.^48^ Coherently with the reported scenario, the degradation kinetics of CYN that mostly reacts with ^•^OH would not change significantly as water evaporates (a similar behavior is also expected for the reaction between CYN and CO_3_^•–^),^48^ while MC-LR photodegradation would accelerate.

Reaction kinetics are known to be affected by temperature, as an increase in temperature accelerates reaction rates.^49,50^ However, in the case of MC-LR and CYN, the reaction rate constants *k*_MC-LR+^•^OH_, *k*_MC-LR+^3^CDOM*_, and *k*_CYN+^•^OH_ are quite high, which means that these reactions have low activation energies. Therefore, the effect of temperature on the indirect photochemical degradation of MC-LR and CYN is expected to be small, especially in the case of MC-LR where other environmental parameters would play a more important role.

## 5 Environmental implications

As the planet continues to experience climate change, the frequency of HABs will continue to increase, and also, the geographical distribution of these events will expand as the temperature increases. Although significant work has been dedicated to understanding different aspects of cyanotoxin formation and occurrence, more emphasis is needed on understanding the natural degradation pathways for these compounds.

Here, we discuss how different scenarios that are impacted by climate change may affect photodegradation of the only two cyanotoxins for which photochemical kinetics is known in sufficient detail, namely, MC-LR and CYN. Faster photodegradation of MC-LR in the presence of water browning or evaporation will at least partially offset its more widespread occurrence. Enhanced photodegradation is predicted for MC-LR because it reacts significantly with ^3^CDOM*, while no important changes are expected for CYN that mostly reacts with ^•^OH (and, additionally, with CO_3_^•–^). Very little is currently known about the reactivity of other cyanotoxins with ^3^CDOM*, which is a major knowledge gap when trying to determine the possible future evolution of cyanotoxin photodegradation in freshwater.

This perspective only considers freshwater scenarios. In the case of seawater, the role of ^•^OH in photodegradation is decreased by its efficient scavenging by Br^–^. The scavenging process yields halogen radicals like Br_2_^•–^ and BrCl^•–^, which are also known to cause cyanotoxin degradation.^51^ However, the large water mass of the ocean responds very slowly to climate change, differently from freshwater lakes that have much smaller thermal capacity.^52^ Therefore, important climate-related changes are probably not foreseen for the photodegradation kinetics of cyanotoxins in seawater.

## References

[ref1] O’Neil J. M.; Davis T. W.; Burford M. A.; Gobler C. J. The rise of harmful cyanobacteria blooms: The potential roles of eutrophication and climate change. Harmful Algae 2012, 14, 313–334. 10.1016/j.hal.2011.10.027.

[ref2] Sellner K. G.; Doucette G. J.; Kirkpatrick G. J. Harmful algal blooms: causes, impacts and detection. J. Ind. Microbiol. Biotechnol. 2003, 30, 383–406. 10.1007/s10295-003-0074-9. 12898390

[ref3] Nelson N. G.; Muñoz-Carpena R.; Phlips E. J.; Kaplan D.; Sucsy P.; Hendrickson J. Revealing biotic and abiotic controls of harmful algal blooms in a shallow subtropical lake through statistical machine learning. Environ. Sci. Technol. 2018, 52, 3527–3535. 10.1021/acs.est.7b05884. 29478313

[ref4] Wu X.; Noss C.; Liu L.; Lorke A. Effects of small-scale turbulence at the air-water interface on microcystis surface scum formation. Water Res. 2019, 167, 115091 10.1016/j.watres.2019.115091. 31561089

[ref5] Lehman P. W.; Teh S. J.; Boyer G. L.; Nobriga M. L.; Bass E.; Hogle C. Initial impacts of Microcystis aeruginosa blooms on the aquatic food web in the San Francisco Estuary. Hydrobiologia 2010, 637, 229–248. 10.1007/s10750-009-9999-y.

[ref6] Wilkinson A. A.; Hondzo M.; Guala M. Investigating abiotic drivers for vertical and temporal heterogeneities of cyanobacteria concentrations in lakes using a seasonal in situ monitoring station. Water Resour. Res. 2019, 55, 954–972. 10.1029/2018WR024228.

[ref7] Scheffer M.; Rinaldi S.; Gragnani A.; Mur L. R.; van Nes E. H. On the dominance of filamentous cyanobacteria in shallow, turbid lakes. Ecology 1997, 78, 272–282. 10.1890/0012-9658(1997)078[0272:OTDOFC]2.0.CO;2.

[ref8] Agnihotri V. K. Anabaena flos-aquae. Crit. Rev. Environ. Sci. Technol. 2014, 44, 1995–2037. 10.1080/10643389.2013.803797.

[ref9] Horppila J.; Holmroos H.; Niemistö J.; Massa I.; Nygrén N.; Schönach P.; Tapio P.; Tammeorg O. Variations of internal phosphorus loading and water quality in a hypertrophic lake during 40 years of different management efforts. Ecol. Engineer. 2017, 103, 264–274. 10.1016/j.ecoleng.2017.04.018.

[ref10] Lu J.; Zhu B.; Struewing I.; Xu N.; Duan S. Nitrogen–phosphorus-associated metabolic activities during the development of a cyanobacterial bloom revealed by metatranscriptomics. Sci. Rep. 2019, 9, 2480 10.1038/s41598-019-38481-2. 30792397 PMC6385219

[ref11] Degerholm J.; Gundersen K.; Bergman B.; Söderbäck E. Phosphorus-limited growth dynamics in two Baltic Sea cyanobacteria, Nodularia sp. and Aphanizomenon sp. FEMS Microbiol. Ecol. 2006, 58, 323–332. 10.1111/j.1574-6941.2006.00180.x. 17117977

[ref12] Paerl H. W.; Paul V. J. Climate change: Links to global expansion of harmful cyanobacteria. Water Res. 2012, 46, 1349–1363. 10.1016/j.watres.2011.08.002. 21893330

[ref13] Ger K. A.; Faassen E. J.; Pennino M. G.; Lürling M. Effect of the toxin (microcystin) content of Microcystis on copepod grazing. Harmful Algae 2016, 52, 34–45. 10.1016/j.hal.2015.12.008. 28073469

[ref14] Trenberth K. E. Changes in precipitation with climate change. Clim. Res. 2011, 47, 123–138. 10.3354/cr00953.

[ref15] Myhre G.; Alterskjær K.; Stjern C. W.; Hodnebrog Ø.; Marelle L.; Samset B. H.; Sillmann J.; Schaller N.; Fischer E.; Schulz M.; Stohl A. Frequency of extreme precipitation increases extensively with event rareness under global warming. Sci. Rep. 2019, 9, 16063 10.1038/s41598-019-52277-4. 31690736 PMC6831572

[ref16] Panton A.; Couceiro F.; Fones G. R.; Purdie D. A. The impact of rainfall events, catchment characteristics and estuarine processes on the export of dissolved organic matter from two lowland rivers and their shared estuary. Sci. Total Environ. 2020, 735, 139481 10.1016/j.scitotenv.2020.139481. 32473434

[ref17] Withers P. J. A.; Lord E. I. Agricultural nutrient inputs to rivers and groundwaters in the UK: policy, environmental management and research needs. Sci. Total Environ. 2002, 282–283, 9–24. 10.1016/S0048-9697(01)00935-4. 11852908

[ref18] Gehringer M. M.; Wannicke N. Climate change and regulation of hepatotoxin production in Cyanobacteria. FEMS Microbiol. Ecol. 2014, 88, 1–25. 10.1111/1574-6941.12291. 24490596

[ref19] Janssen E. M. L. Cyanobacterial peptides beyond microcystins – A review on co-occurrence, toxicity, and challenges for risk assessment. Water Res. 2019, 151, 488–499. 10.1016/j.watres.2018.12.048. 30641464

[ref20] Kurtz T.; Zeng T.; Rosario-Ortiz F. L. Photodegradation of cyanotoxins in surface waters. Water Res. 2021, 192, 116804 10.1016/j.watres.2021.116804. 33494040

[ref21] Song W.; Yan S.; Cooper W. J.; Dionysiou D. D.; O’Shea K. E. Hydroxyl radical oxidation of cylindrospermopsin (cyanobacterial toxin) and its role in the photochemical transformation. Environ. Sci. Technol. 2012, 46, 12608–12615. 10.1021/es302458h. 23082747

[ref22] van Apeldoorn M. E.; van Egmond H. P.; Speijers G. J.; Bakker G. J. Toxins of cyanobacteria. Mol. Nutr. Food Res. 2007, 51, 7–60. 10.1002/mnfr.200600185. 17195276

[ref23] Bláha L.; Babica P.; Maršálek B. Toxins produced in cyanobacterial water blooms - toxicity and risks. Interdiscip. Toxicol. 2009, 2, 36–41. 10.2478/v10102-009-0006-2. 21217843 PMC2984099

[ref24] Tang T.; Hoefel D.; Mosisch T.; Ho L. Assessing the fate and biodegradation of cyanobacterial metabolites in Australian waters. Water Pract. Technol. 2012, 7, wpt2012064 10.2166/wpt.2012.064.

[ref25] Minella M.; Leoni B.; Salmaso N.; Savoye L.; Sommaruga R.; Vione D. Long-term trends of chemical and modelled photochemical parameters in four Alpine lakes. Sci. Total Environ. 2016, 541, 247–256. 10.1016/j.scitotenv.2015.08.149. 26410700

[ref26] Vione D.; Scozzaro A. Photochemistry of surface fresh waters in the framework of climate change. Environ. Sci. Technol. 2019, 53, 7945–7963. 10.1021/acs.est.9b00968. 31241909

[ref27] Butcher J. B.; Nover D.; Johnson T. E.; Clark C. M. Sensitivity of lake thermal and mixing dynamics to climate change. Clim. Change 2015, 129, 295–305. 10.1007/s10584-015-1326-1.

[ref28] Remucal C. K. The role of indirect photochemical degradation in the environmental fate of pesticides: a review. Environ. Sci.: Processes Impacts 2014, 16, 628–653. 10.1039/c3em00549f. 24419250

[ref29] Yan S.; Song W. Photo-transformation of pharmaceutically active compounds in the aqueous environment: a review. Environ. Sci. Process Impacts 2014, 16, 697–720. 10.1039/C3EM00502J. 24608883

[ref30] Rosario-Ortiz F. L.; Canonica S. Probe compounds to assess the photochemical activity of dissolved organic matter. Environ. Sci. Technol. 2016, 50, 12532–12547. 10.1021/acs.est.6b02776. 27736067

[ref31] Vione D. A critical view of the application of the APEX software (Aqueous Photochemistry of Environmentally-occurring Xenobiotics) to predict photoreaction kinetics in surface freshwaters. Molecules 2020, 25, 9 10.3390/molecules25010009. PMC7017383 31861417

[ref32] Braslavsky S. E. Glossary of terms used in photochemistry. third edition. Pure Appl. Chem. 2007, 79, 293–465. 10.1351/pac200779030293.

[ref33] Williamson C. E.; Overholt E. P.; Pilla R. M.; Leach T. H.; Brentrup J. A.; Knoll L. B.; Mette E. M.; Moeller R. E. Ecological consequences of long-term browning in lakes. Sci. Rep. 2016, 5, 18666 10.1038/srep18666. PMC4687041 26690504

[ref34] Weyhenmeyer G. A.; Müller R. A.; Norman M.; Tranvik L. J. Sensitivity of freshwaters to browning in response to future climate change. Clim. Change 2016, 134, 225–239. 10.1007/s10584-015-1514-z.

[ref35] Solomon C. T.; Jones S. E.; Weidel B. C.; Buffam I.; Fork M. L.; Karlsson J.; Larsen S.; Lennon J. T.; Read J. S.; Sadro S.; Saros J. E. Ecosystem consequences of changing inputs of terrestrial dissolved organic matter to lakes: Current knowledge and future challenges. Ecosystems 2015, 18, 376–389. 10.1007/s10021-015-9848-y.

[ref36] Minella M.; De Laurentiis E.; Buhvestova O.; Haldna M.; Kangur K.; Maurino V.; Minero C.; Vione D. Modelling lake-water photochemistry: three-decade assessment of the steady-state concentration of photoreactive transients (^•^OH, CO_3_^•-^ and ^3^CDOM*) in the surface water of polymictic Lake Peipsi (Estonia/Russia). Chemosphere 2013, 90, 2589–2596. 10.1016/j.chemosphere.2012.10.103. 23273735

[ref37] Calderaro F.; Vione D. Possible effect of climate change on surface-water photochemistry: A model assessment of the impact of browning on the photodegradation of pollutants in lakes during summer stratification. Epilimnion vs. whole-lake phototransformation. Molecules 2020, 25, 2795 10.3390/molecules25122795. PMC7356553 32560420

[ref38] He X.; de la Cruz A. A.; Hiskia A.; Kaloudis T.; O’Shea K.; Dionysiou D. D. Destruction of microcystins (cyanotoxins) by UV-254nm-based direct photolysis and advanced oxidation processes (AOPs): Influence of variable amino acids on the degradation kinetics and reaction mechanisms. Water Res. 2015, 74, 227–238. 10.1016/j.watres.2015.02.011. 25744186

[ref39] Yan S.; Zhang D.; Song W. Mechanistic considerations of photosensitized transformation of microcystin-LR (cyanobacterial toxin) in aqueous environments. Environ. Pollut. 2014, 193, 111–118. 10.1016/j.envpol.2014.06.020. 25016104

[ref40] Halladja S.; Ter Halle A.; Aguer J. P.; Boulkamh A.; Richard C. Inhibition of humic substances mediates photooxigenation of furfuryl alcohol by 2,4,6-trimethylphenol. Evidence for reactivity of the phenol with humic triplet excited states. Environ. Sci. Technol. 2007, 41, 6066–6073. 10.1021/es070656t. 17937283

[ref41] Al Housari F.; Vione D.; Chiron S.; Barbati S. Reactive photoinduced species in estuarine waters. Characterization of hydroxyl radical, singlet oxygen and dissolved organic matter triplet state in natural oxidation processes. Photochem. Photobiol. Sci. 2010, 9, 78–86. 10.1039/B9PP00030E. 20062847

[ref42] He X.; Delacruz A.; Dionysiou D. Destruction of cyanobacterial toxin cylindrospermopsin by hydroxyl radicals and sulfate radicals using UV-254 nm activation of hydrogen peroxide, persulfate and peroxymonosulfate. J. Photochem. Photobiol., A 2013, 251, 160–166. 10.1016/j.jphotochem.2012.09.017.

[ref43] Onstad G. D.; Strauch S.; Meriluoto J.; Codd G. A.; Von Gunten U. Selective oxidation of key functional groups in cyanotoxins during drinking water ozonation. Environ. Sci. Technol. 2007, 41, 4397–4404. 10.1021/es0625327. 17626442

[ref44] Hao Z.; Ma J.; Miao C.; Song Y.; Lian L.; Yan S.; Song W. Carbonate radical oxidation of cylindrospermopsin (cyanotoxin): Kinetic studies and mechanistic consideration. Environ. Sci. Technol. 2020, 54, 10118–10127. 10.1021/acs.est.0c03404. 32693577

[ref45] Canonica S.; Kohn T.; Mac M.; Real F. J.; Wirz J.; Von Gunten U. Photosensitizer method to determine rate constants for the reaction of carbonate radical with organic compounds. Environ. Sci. Technol. 2005, 39, 9182–9188. 10.1021/es051236b. 16382940

[ref46] Mosley L. M.; Zammit B.; Leyden E.; Heneker T. M.; Hipsey M. R.; Skinner D.; Aldridge K. T. The impact of extreme low flows on the water quality of the Lower Murray River and Lakes (South Australia). Water Resour. Manag. 2012, 26, 3923–3946. 10.1007/s11269-012-0113-2.

[ref47] Mosley L. M. Drought impacts on the water quality of freshwater systems; review and integration. Earth-Sci. Rev. 2015, 140, 203–214. 10.1016/j.earscirev.2014.11.010.

[ref48] Carena L.; Terrenzio D.; Mosley L. M.; Toldo M.; Minella M.; Vione D. Photochemical consequences of prolonged hydrological drought: A model assessment of the Lower Lakes of the Murray-Darling Basin (Southern Australia). Chemosphere 2019, 236, 124356 10.1016/j.chemosphere.2019.124356. 31330437

[ref49] McKay G.; Dong M. M.; Kleinman J. L.; Mezyk S. P.; Rosario-Ortiz F. L. Temperature dependence of the reaction between the hydroxyl radical and organic matter. Environ. Sci. Technol. 2011, 45, 6932–6937. 10.1021/es201363j. 21662387

[ref50] Kieber D. J.; Miller G. W.; Neale P. J.; Mopper K. Wavelength and temperature-dependent apparent quantum yields for photochemical formation of hydrogen peroxide in seawater. Environ. Sci. Process Impacts. 2014, 16, 777–791. 10.1039/C4EM00036F. 24615241

[ref51] Parker K. M.; Mitch W. A. Halogen radicals contribute to photooxidation in coastal and estuarine waters. Proc. Natl. Acad. Sci. U. S. A. 2016, 113, 5868–5873. 10.1073/pnas.1602595113. 27162335 PMC4889391

[ref52] Adrian R.; O’Reilly C. M.; Zagarese H.; Baines S. B.; Hessen D. O.; Keller W.; Livingstone D. M.; Sommaruga R.; Straile D.; Van Donk E.; Weyhenmeyer G. A.; Winder M. Lakes as sentinels of climate change. Limnol. Oceanogr. 2009, 54, 2283–2297. 10.4319/lo.2009.54.6_part_2.2283. 20396409 PMC2854826

